# Association between urinary albumin-to-creatinine ratio and all-cause and cardiovascular-cause mortality among MASLD: NHANES 2001–2018

**DOI:** 10.3389/fnut.2025.1528732

**Published:** 2025-05-09

**Authors:** Zhengjin Wang, Zhangxin Chen, Hanxu Zhuang

**Affiliations:** ^1^Zhangzhou Health Vocational College, Zhangzhou, Fujian Province, China; ^2^Department of Spine Surgery, Renmin Hospital of Wuhan University, Wuhan, Hubei Province, China; ^3^Department of Gastroenterology, Zhangzhou Affiliated Hospital of Fujian Medical University, Zhangzhou, Fujian Province, China

**Keywords:** UACR, MASLD, all-cause mortality, cardiovascular mortality, NHANES

## Abstract

**Background:**

Urinary albumin-to-creatinine ratio (UACR) is an established biomarker for assessing kidney damage, but recent studies suggest it may also reflect broader health risks. This study aimed to investigate the association between UACR and all-cause and cardiovascular disease (CVD)-cause mortality in patients with metabolic dysfunction-associated steatotic liver disease (MASLD).

**Methods:**

In this prospective cohort study, we included sample of 3,412 MASLD enrolled in the National Health and Nutrition Examination Survey 2001–2018. The study population was divided into three different risk categories based on urinary UACR: low level (<4.67 mg/g), intermediate level (4.67–7.67 mg/g), and high level (7.68–30 mg/g). Cox proportional hazards models were used to estimate the hazard ratios (HR) for the association between UACR level and both all-cause and CVD-cause mortality. Restricted cubic spline (RCS) curve analysis was employed to assess the non-linear association between UACR and mortality. Kaplan-Meier (KM) survival curves were used to evaluate survival rates across UACR groups.

**Results:**

The study found that higher UACR levels, even within the normal range, were independently associated with increased risks of both all-cause and CVD-cause mortality. Each 1 mg/g increase in UACR was associated with a 4% higher risk of all-cause mortality (HR 1.04, 95% CI 1.03–1.05) and a 5% higher risk of cardiovascular mortality (HR 1.05, 95% CI 1.02–1.08). Compared with the low UACR group, high UACR both showed an increased all-cause mortality risk [HR, 2.69 (95% CI, 2.07–3.50)] and CVD-cause mortality risk [HR, 2.97 (95% CI, 1.76–4.99)]. RCS curve analysis revealed a non-linear positive correlation between UACR and both all-cause and CVD-cause mortality, identifying UACR thresholds of 7.467 mg/g for all-cause mortality and 7.195 mg/g for CVD-cause mortality. The KM survival curves confirmed that participants with lower UACR levels had higher survival rates.

**Conclusion:**

Elevated UACR levels within the normal range, are associated with increased all-cause and cardiovascular mortality in patients with MASLD. UACR may serve as a useful early biomarker for identifying individuals at higher risk of mortality, supporting more proactive clinical interventions to manage MASLD-related risks.

## 1 Introduction

Metabolic dysfunction-associated steatotic liver disease (MASLD) is a chronic liver disease characterized by hepatic fat accumulation and has now become a leading cause of chronic liver disease worldwide (1–3). The development of MASLD is closely associated with various metabolic disorders, including obesity, insulin resistance, and dyslipidemia (1). Although classified as a hepatic disorder, MASLD is now recognized as a multisystem disorder with significant implications for cardiovascular, renal, and endocrine systems. Multiple studies have demonstrated that MASLD is a significant risk factor for chronic kidney disease (CKD) and is closely associated with elevated urinary albumin levels, suggesting a potential link between hepatic and renal metabolic dysfunction (4–8).

The urinary albumin-to-creatinine ratio (UACR) is a sensitive biomarker for assessing renal filtration function and quantifying albuminuria, demonstrating particular value in the early detection of microalbuminuria (9). In clinical practice, a UACR >30 mg/g is a key threshold, often indicating the presence of kidney damage, particularly in patients with diabetes. Numerous researches have demonstrated that elevated UACR levels are associated with an increased risk of cardiovascular disease (CVD) and serve as important predictors of cardiovascular mortality in individuals with hypertension and diabetes (10, 11). Interestingly, even high-normal UACR levels within the reference range have been linked to a significantly higher risk of all-cause mortality (12). Given that both metabolic MASLD and CKD are established risk factors for CVD and cardiovascular death (13, 14), and considering that MASLD itself may contribute to renal impairment. Therefore, monitoring UACR may offer important clinical value for health assessment in patients with MASLD. However, the role of UACR in assessing mortality risk among MASLD patients remains poorly understood, particularly whether elevated levels of UACR in the normal range also increase the risk of death.

This study analyzed data from the National Health and Nutrition Examination Survey (NHANES) and the National Death Index (NDI) to investigate whether UACR could serve as a biomarker for assessing cardiovascular disease risk and all-cause mortality in patients with MASLD.

## 2 Methods

### 2.1 Data source and study population

**Figure 1:**
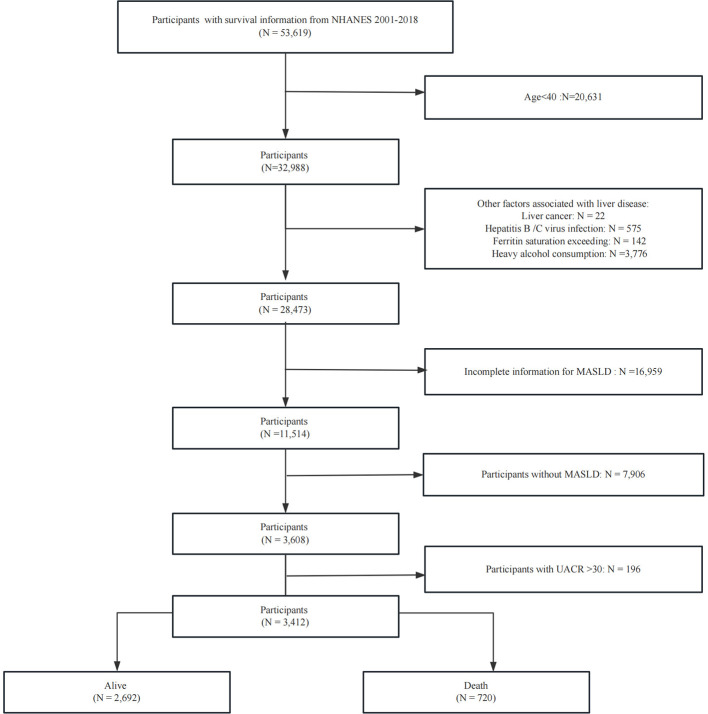
Flow chart of sample selection from the NHANES.

The NHANES is a nationally representative, cross-sectional study designed to evaluate the health and nutritional status of the US population. It employs a standardized protocol integrating detailed household interviews (capturing demographic, dietary, and health-related data), comprehensive physical examinations in mobile examination centers (MECs), and advanced laboratory testing of biological specimens. The analysis utilized data collected across nine survey cycles between 2001 and 2018. We excluded specific groups from our analysis to ensure a focused study population: (1) Age <40 years; (2) individuals with liver cancer; (3) individuals with high alcohol intake, defined as more than three alcoholic beverages per day for men and more than two per day for women; (4) individuals diagnosed with hepatitis B or C, identified by positive hepatitis B surface antigen or hepatitis C antibody/HCV RNA tests; and (5) individuals with ferritin saturation levels exceeding 50%. (6) Lack of information to evaluate MASLD; (7) individuals with UACR >30. Figure 1 provides a visual representation of the patient selection process.

### 2.2 Definition of MASLD

The diagnosis of MASLD requires both evidence of hepatic steatosis and at least one metabolic risk factor (e.g., obesity, diabetes, or metabolic syndrome components), while excluding other liver diseases and excessive alcohol consumption. Liver biopsy is the gold standard for diagnosing hepatic steatosis, but due to its limitations such as invasiveness, non-invasive methods are preferred in clinical practice (15, 16). Currently, Controlled attenuation parameter techniques based on vibration controlled transient elastography have become an important alternative diagnostic tool due to their high accuracy and ability to simultaneously assess fibrosis. However, this study was limited by the availability of liver ultrasound data in most survey cycles of NHANES, so the US Fatty Liver Index (USFLI) was used to define MASLD. This methodological choice was based on previous validation studies confirming that a USFLI score ≥30 as a cutoff has reasonable diagnostic efficacy (AUROC of 0.80–0.85 compared to MRI-confirmed steatosis) (17, 18).

### 2.3 Definition of UACR

Urinary albumin and creatinine levels were used to calculate the UACR. To further investigate potential differences within the normal range of UACR levels, we categorized participants with UACR <30 mg/g into three subgroups based on previous studies (12): low (<4.67 mg/g), intermediate (4.67–7.67 mg/g), and high (7.68–30 mg/g) level groups.

### 2.4 Ascertainment of mortality

The primary endpoints of this study were all-cause and cardiovascular disease (CVD)-specific mortality, as determined by records from the National Death Index (NDI) up to December 31, 2019. All-cause mortality includes deaths from heart disease, malignant neoplasms, nephritis, nephrotic syndrome and nephrosis, Alzheimer's disease, diabetes mellitus, chronic lower respiratory diseases, influenza and pneumonia, cerebrovascular diseases, accidents (unintentional injuries), and other causes. CVD-cause mortality as deaths due to cardiovascular disease. Further details on these classifications are provided in a previous study (19).

### 2.5 Assessment of covariates

In this study, we adjusted for potential confounders that could influence the outcomes. Demographic characteristics were collected through standardized questionnaires during household interviews, including age, gender, race (Mexican American, White, Black, and other races), poverty status, education level (below high school, high school, and above high school), smoking status and alcohol assumption. Relevant laboratory measures, including liver function tests (ALT, AST) blood albumin, blood urea nitrogen, creatinine, and estimated glomerular filtration rate (eGFR), were obtained through mobile testing centers. Additionally, we collected information on the presence of hypertension (yes/no), diabetes (yes/no), and chronic kidney disease (yes/no), and CVD (yes/no; The above detailed definitions are attached in the Supplementary file).

### 2.6 Statistical analysis

This study included 3,412 participants, who were categorized into three groups based on UACR. For variables with missing values, we conducted multiple imputations based on “mice” package. Descriptive statistics were presented for both categorical and continuous variables. For continuous variables, the one-way analysis of variance was used to compare multiple samples. Categorical data were expressed as frequencies (*n*, %) and analyzed using the Chi-square test, with *P* < 0.05 considered statistically significant. Multiple Cox proportional hazards regression models were used to examine the association between UACR and the risk of all-cause mortality and CVD-cause mortality across three analytical models. Crude Model was adjusted for age, gender, ethnicity, poverty, Model 1 adjusted for, blood urea nitrogen, albumin eGFR, SII Alt, and Ast on the basis of Crude Model., and Model 2 included additional variables, including smoking status, history of hypertension, and diabetes, and CVD. Hazard ratios (HR) and corresponding 95% confidence intervals (CI) were calculated. Kaplan-Meier (KM) survival analysis was employed to compare all-cause and cardiovascular mortality risks across the three UACR. Additionally, a restricted cubic spline (RCS) regression model was employed to assess the linear relationship between continuous UACR levels and risk of death. Subgroup analyses were conducted, and interactions within subgroups were evaluated using the likelihood ratio test. Analyses were conducted using NHANES-recommended sampling weights to account for the complex survey design. Statistical analysis was performed using R Statistical Software (Version 4.3.3), with statistical significance defined as a *P*-value < 0.05. The study was approved by the National Center for Health Statistics Ethics Review Board, and all participants provided informed consent.

## 3 Result

### 3.1 Baseline characteristics of the participants

The baseline characteristics of the cohort, classified according to UACR levels, are shown in Table 1. The average age of the participants was 59.58 years, with 57.44% being male and 76.08% identifying as White. The prevalence of hypertension and diabetes was 64.29% and 37.69%, respectively. Compared to the low level of UACR, participants with the high level of UACR were older, had higher SII levels, and were more likely to have a history of hypertension, diabetes, and CVD. In contrast, the high level of UACR group had lower educational levels, higher poverty rates. There were no significant differences in BMI, Ast, blood urea nitrogen, blood albumin and smoking among the three groups. A total of 720 (15.93%) deaths occurred during the follow-up period, 238 of which were attributed to CVD. Participants in the group with high UACR levels had a higher mortality rate than those in the group with low UACR levels.

**Table 1:** Baseline characteristics of the participants.

**Variable**	**Total, *n* = 3,412**	**Low,** ***n* = 726**	**Intermediate, *n* = 1,096**	**High,** ***n* = 1,590**	***P-value***
Age (year)	59.58 (0.29)	56.03 (0.48)	58.89 (0.45)	62.48 (0.47)	<0.0001
BMI (kg/m^2^)	33.61 (0.17)	33.44 (0.32)	33.70 (0.27)	33.65 (0.27)	0.815
eGFR (mL/min/1.73)	82.78 (0.43)	83.38 (0.76)	84.03 (0.70)	81.36 (0.78)	0.031
Alt (IU/L)	28.73 (0.36)	30.29 (0.69)	28.96 (0.62)	27.51 (0.55)	0.009
Ast (IU/L)	25.94 (0.24)	26.00 (0.51)	26.01 (0.41)	25.84 (0.37)	0.943
PIR	3.12 (0.05)	3.47 (0.09)	3.15 (0.07)	2.87 (0.06)	<0.0001
Blood urea nitrogen (mg/dL)	15.45 (0.14)	15.27 (0.24)	15.01 (0.24)	15.93 (0.28)	0.051
Blood Albumin (g/dL)	4.16 (0.01)	4.19 (0.02)	4.14 (0.01)	4.17 (0.01)	0.125
SII	566.64 (6.89)	533.30 (12.93)	560.40 (9.86)	593.63 (11.48)	0.002
**Sex (%)**					<0.0001
Female	1,552 (42.56)	208 (24.53)	520 (44.25)	824 (53.05)	
Male	1,860 (57.44)	518 (75.47)	576 (55.75)	766 (46.95)	
**Race (%)**					0.074
White	1,681 (76.08)	380 (79.39)	537 (75.74)	764 (74.20)	
Black	400 (5.62)	103 (5.80)	121 (5.01)	176 (6.01)	
Mexican	739 (7.93)	138 (6.28)	236 (8.24)	365 (8.76)	
Other	592 (10.36)	105 (8.53)	202 (11.01)	285 (11.04)	
**Education (%)**					<0.0001
Less than high school	620 (8.64)	98 (6.16)	184 (7.90)	338 (10.87)	
High school	1,277 (36.52)	257 (31.34)	408 (36.44)	612 (40.00)	
Some college or AA degree	1,515 (54.84)	371 (62.50)	504 (55.66)	640 (49.13)	
**Smoking (%)**					0.866
Former	1,194 (36.07)	248 (35.48)	363 (35.65)	583 (36.80)	
Never	1,792 (51.62)	379 (53.12)	593 (51.79)	820 (50.49)	
Now	426 (12.31)	99 (11.39)	140 (12.56)	187 (12.71)	
**Hypertension (%)**					<0.0001
No	1,151 (35.71)	312 (44.00)	390 (35.48)	449 (30.44)	
Yes	2,261 (64.29)	414 (56.00)	706 (64.52)	1,141 (69.56)	
**Diabetes (%)**					<0.0001
No	1,889 (62.31)	495 (73.55)	642 (64.47)	752 (53.18)	
Yes	1,523 (37.69)	231 (26.45)	454 (35.53)	838 (46.82)	
**CVD (%)**					<0.001
No	2,696 (80.93)	606 (86.47)	880 (81.61)	1,210 (76.74)	
Yes	716 (19.07)	120 (13.53)	216 (18.39)	380 (23.26)	
**Survival status (%)**					<0.0001
Alive	2,692 (84.07)	643 (92.57)	915 (86.02)	1,134 (76.89)	
Death	720 (15.93)	83 (7.43)	181 (13.98)	456 (23.11)	

BMI, Body Mass Index; PIR, poverty income ratio; SII, systemic immune inflammatory index; CVD, cardiovascular disease.

### 3.2 Association of UACR level with the risk of all-cause death in patients with MASLD

**Figure 2:**
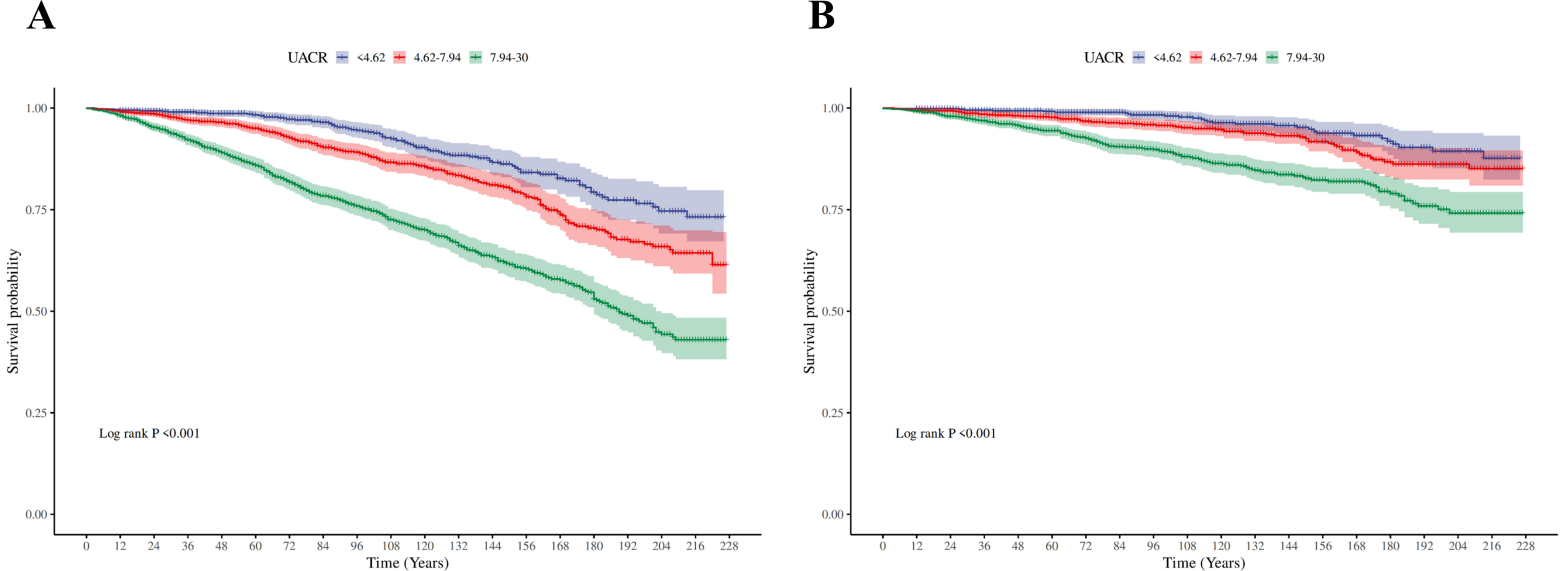
Kaplan–Meier (KM) survival curves. **(A)** The Kaplan-Meier curves for three different groups (for all-cause death). **(B)** The Kaplan-Meier curves for three different groups (for CVD-cause death).

**Figure 3:**
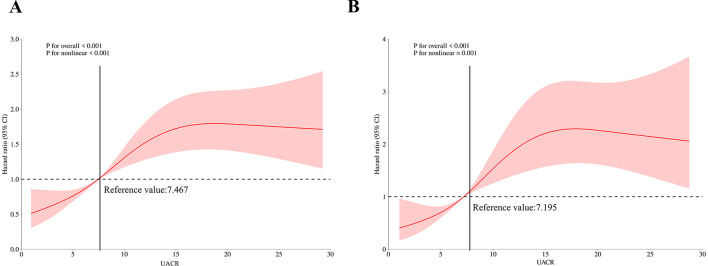
Restricted cubic spline curves of relations between UACR and death. **(A)** Restricted cubic spline curves of relations between UACR with all-cause death risk; **(B)** Restricted cubic spline curves of relations between UACR with CVD-cause death risk.

Table 2 presents the relationship between UACR and Risk of death in patients with MASLD. In fully adjusted multivariable Cox models, each 1 mg/g increment in UACR was associated with a statistically significant 4% increase in all-cause mortality risk (HR 1.04, 95% CI 1.03–1.05). When categorized by UACR risk levels and in the fully adjusted model (Model 3), participants with intermediate level of UACR showed 81% higher mortality risk (HR 1.81, 95% CI 1.32,2.48; *P* < 0.001), and participants with high level of UACR showed 169% higher mortality risk (HR 2.69, 95% CI 2.07,3.50; *P* < 0.001), compared with the low level of UACR group. These findings were consistent across the three models (Model 1, Model 2, and Model 3). The KM curve further indicated that participants with low level of UACR had a survival advantage over those with higher UACR levels (Figure 2A). The RCS regression demonstrated a non-linear association between UACR and all-cause mortality, displaying a distinct inverted L-shaped pattern (Figure 3A). When the level of UACR >7.467, the risk of all-cause death was significantly increased.

**Table 2:** Association of UACR with the risk of death in MASLD.

	**Crude model**		**Model 1**		**Model 2**	
	**95%CI**	***P***	**HR (95%CI)**	***P***	**HR (95%CI)**	***P***
**All cause death**						
**UACR**	1.05 (1.03, 1.06)	<0.0001	1.05 (1.03, 1.06)	<0.0001	1.04 (1.03, 1.05)	<0.0001
Low	ref		ref		ref	
Intermediate	1.77 (1.28, 2.43)	<0.001	1.81 (1.32, 2.47)	<0.001	1.81 (1.32, 2.48)	<0.001
High	2.66 (2.01, 3.51)	<0.0001	2.80 (2.13, 3.68)	<0.0001	2.69 (2.07, 3.50)	<0.0001
***p*** **for trend**		<0.01		<0.01		<0.01
**CVD-cause death**						
**UACR**	1.05 (1.03, 1.08)	<0.0001	1.06 (1.03, 1.08)	<0.0001	1.05 (1.02, 1.08)	<0.001
Low	ref		ref		ref	
Intermediate	2.12 (1.20, 3.75)	0.01	2.13 (1.23, 3.67)	0.01	2.02 (1.14, 3.57)	0.02
High	2.89 (1.68, 4.96)	<0.001	3.17 (1.87, 5.36)	<0.0001	2.97 (1.76, 4.99)	<0.0001
***p*** **for trend**		<0.05		<0.05		<0.05

Crude Model: adjusted for age, gender, ethnicity, poverty.

Model 1: adjusted for, blood urea nitrogen, albumin eGFR, SII Alt, and Ast on the basis of Crude Model.

Model 2: Additionally adjust for smoking status, hypertension, and diabetes, and CVD on the basis of Model 1.

UACR, Urinary albumin-to-creatinine ratio; CVD, cardiovascular disease; SII, systemic immune inflammatory index; MASLD, metabolic dysfunction-associated steatotic liver disease.

### 3.3 Association of UACR with the risk of CVD-cause death in patients with MASLD

For CVD-cause mortality, a similar pattern was observed for relationship between UACR and CVD-cause death (Table 2). In multivariable-adjusted models, each 1 mg/g increase in UACR was associated with a 5% higher risk of CVD-cause death (HR 1.05, 95% CI 1.02–1.08). When grouped by UACR risk levels in the fully adjusted model (Model 3), compared with the low level of UACR group, both the intermediate level of UACR group [HR, 2.02 (95% CI, 1.14, 3.57), *P* < 0.05] and high level of UACR group [HR, 2.97 (95% CI, 1.76, 4.99), *P* < 0.0001] had a significantly higher risk of CVD-cause death. The KM curve further indicated that participants with low level of UACR had a survival advantage over those with higher UACR levels (Figure 2B). The relationship between UACR and CVD-cause mortality was also non-linear and inverted L-shaped based on the RCS model (Figure 3B). When the level of UACR >7.195, the risk of CVD-cause death was significantly increased.

### 3.4 Subgroup analyses

Subgroup analyses assessing the stability of the association between UACR and mortality (including both all-cause and CVD-cause deaths) are presented in Tables 3, 4. The trends in the effects of UACR on mortality were consistent with those observed in the overall cohort. No significant interactions were found between UACR and the risk of death across the stratified variables (all p-interactions > 0.05).

**Table 3:** Subgroup analysis of the association between UACR level and all-cause mortality among patients with MASLD.

**Character**	**Low**	**Intermediate**	***p***	**High**	***p***	***p* for trend**	***p* for interaction**
**Sex**							0.582
Female	ref	2.05 (1.37, 3.07)	<0.001	4.46 (3.22, 6.16)	<0.0001	<0.001	
Male	ref	2.94 (1.61, 5.36)	<0.001	5.02 (2.65, 9.52)	<0.0001	<0.0001	
**Race**							0.115
White	ref	2.37 (1.62, 3.45)	<0.0001	4.21 (3.04, 5.83)	<0.0001	<0.0001	
Mexican	ref	1.02 (0.48, 2.15)	0.965	2.78 (1.42, 5.44)	0.003	0.002	
Black	ref	2.06 (0.97, 4.38)	0.061	3.60 (1.71, 7.60)	<0.001	<0.001	
Other	ref	0.59 (0.20, 1.68)	0.318	1.24 (0.47, 3.26)	0.660	0.238	
**Education**							0.143
High School	ref	1.76 (1.09, 2.84)	0.020	3.18 (2.18, 4.64)	<0.0001	0.119	
Less than high school	ref	1.04 (0.57, 1.92)	0.892	2.11 (1.31, 3.40)	0.002	<0.001	
Some college or AA degree	ref	2.82 (1.61, 4.93)	<0.001	4.49 (2.57, 7.83)	<0.0001	<0.0001	
**Smoking**							0.202
Former	ref	1.63 (1.02, 2.61)	0.041	4.13 (2.79, 6.11)	<0.0001	0.276	
Now	ref	2.06 (0.97, 4.38)	0.060	3.08 (1.52, 6.21)	0.002	0.002	
Never	ref	2.77 (1.55, 4.93)	<0.001	3.92 (2.32, 6.61)	<0.0001	<0.0001	
**Hypertension**							0.724
No	ref	2.38 (1.27, 4.49)	0.007	3.83 (2.25, 6.51)	<0.0001	<0.0001	
Yes	ref	1.88 (1.27, 2.79)	0.002	3.44 (2.45, 4.81)	<0.0001	0.012	
**Diabetes**							0.113
No	ref	2.01 (1.29, 3.14)	0.002	3.98 (2.72, 5.83)	<0.0001	0.009	
Yes	ref	1.88 (1.24, 2.85)	0.003	2.73 (1.85, 4.05)	<0.0001	<0.0001	
**CVD**							0.064
No	ref	2.58 (1.65, 4.03)	<0.0001	3.97 (2.67, 5.93)	<0.0001	<0.001	
Yes	ref	1.10 (0.73, 1.67)	0.650	2.50 (1.63, 3.84)	<0.0001	<0.0001	

UACR, Urinary albumin-to-creatinine ratio; CVD, cardiovascular disease; MASLD, metabolic dysfunction-associated steatotic liver disease.

**Table 4:** Subgroup analysis of the association between UACR level and CVD-cause mortality among patients with MASLD.

**Character**	**Low**	**Intermediate**	***p***	**High**	***p***	***p* for trend**	***p* for interaction**
**Sex**							0.971
Female	ref	2.55 (1.26, 5.16)	0.009	4.92 (2.65, 9.13)	<0.0001	0.008	
Male	ref	3.15 (1.03, 9.65)	0.045	5.77 (1.98, 16.78)	0.001	<0.0001	
**Race**							0.606
White	ref	2.64 (1.42, 4.92)	0.002	4.20 (2.35, 7.51)	<0.0001	<0.0001	
Mexican	ref	0.57 (0.15, 2.17)	0.410	2.50 (0.92, 6.75)	0.071	0.057	
Black	ref	2.46 (0.59, 10.36)	0.218	3.06 (0.79, 11.82)	0.105	0.097	
Other	ref	3.00 (0.72, 12.54)	0.132	7.06 (1.33, 37.55)	0.022	0.621	
**Education**							0.185
High school	ref	1.52 (0.66, 3.47)	0.324	2.42 (1.25, 4.71)	0.009	0.349	
Less than high school	ref	1.40 (0.46, 4.28)	0.558	3.11 (1.32, 7.30)	0.009	0.016	
Some college or AA degree	ref	5.47 (2.28, 13.12)	<0.001	7.94 (2.95, 21.39)	<0.0001	<0.001	
**Smoking**							0.061
Former	ref	1.01 (0.51, 2.01)	0.977	2.94 (1.50, 5.75)	0.002	0.715	
Now	ref	3.54 (0.64, 19.67)	0.148	5.48 (1.01, 29.82)	0.049	0.037	
Never	ref	5.79 (2.16, 15.49)	<0.001	6.90 (2.74, 17.41)	<0.0001	0.469	
**Hypertension**							0.715
Yes	ref	2.59 (1.31, 5.10)	0.006	4.35 (2.33, 8.15)	<0.0001	0.008	
No	ref	1.99 (0.70, 5.65)	0.195	2.42 (0.81, 7.23)	0.113	0.145	
**Diabetes**							0.832
No	ref	2.11 (0.99, 4.48)	0.052	3.34 (1.76, 6.37)	<0.001	0.074	
Yes	ref	2.88 (1.55, 5.38)	<0.001	4.13 (2.22, 7.69)	<0.0001	<0.0001	
**CVD**							0.864
No	ref	2.50 (1.08, 5.79)	0.032	3.82 (1.86, 7.85)	<0.001	0.035	
Yes	ref	1.77 (0.93, 3.36)	0.082	3.08 (1.62, 5.85)	<0.001	0.437	

UACR, Urinary albumin-to-creatinine ratio; CVD, cardiovascular disease; MASLD, metabolic dysfunction-associated steatotic liver disease.

## 4 Discussion

In this prospective cohort study, we explored the association between UACR and both all-cause and CVD-cause mortality in patients with MASLD. The results indicated that higher UACR levels within the normal range, were independently associated with increased mortality in this population. The RCS curves revealed a non-linear, positive correlation between UACR and both all-cause and CVD-cause mortality in MASLD patients. These findings were further confirmed by the KM curves. Our results suggest that UACR may serve as a valuable surrogate biomarker for clinical prognosis in the MASLD population.

Urinary protein excretion is a key biomarker for assessing kidney damage, with a UACR >30 mg/g typically indicating a significant risk of kidney injury. However, recent studies have shown that early signs of kidney and vascular damage may also be present in individuals with UACR levels within the normal range (20). Specifically, several studies in general populations have observed that individuals with higher UACR levels within the normal range exhibit significantly increased risks of all-cause mortality and cardiovascular mortality (12, 21–23). This suggests that even if UACR does not exceed the normal threshold, higher levels may still be associated with adverse health outcomes. Our study supports this perspective by demonstrating that in patients with MASLD, those with higher UACR levels within the normal range have significantly increased risks of all-cause and cardiovascular mortality. In addition, our findings suggest a non-linear relationship between UACR and both all-cause and CVD-cause mortality, with an inverted L-shaped pattern, and identified specific UACR thresholds associated with elevated mortality risks: a threshold of 7.467 mg/g for all-cause mortality and 7.195 mg/g for CVD-cause mortality. However, the exact mechanism by which UACR influences the association between MAFLD and CVD remains unclear. Firstly, elevated UACR reflects increased urinary albumin excretion, which not only serves as a biomarker for kidney disease but also indicates systemic endothelial dysfunction. This endothelial dysfunction is a critical early marker of microvascular damage, which is closely linked to the pathogenesis of CVD. It disrupts the balance of vasodilation and vasoconstriction, increases vascular permeability, and promotes inflammatory and thrombotic processes that contribute to atherosclerosis (24, 25). At the same time, it also means that the kidney's micro-vessels are damaged, leading to tubulointerstitial inflammation and fibrosis, which may further develop into CKD (26). The occurrence of CKD becoming a key factor in the increased risk of CVD. Secondly, Zheng et al.'s research indicates that the SII is positively associated with urinary protein excretion in American adults, suggesting a significant association between UACR and systemic inflammatory response (27). This chronic inflammatory state may not only accelerate liver fibrosis and cirrhosis progression in MASLD but also increase the risk of CVD by exacerbating endothelial damage and atherosclerosis, thereby heightening overall mortality risk. Additionally, studies have shown that elevated UACR may serve as an early warning signal of vascular stiffness, which is significantly associated with an increased risk of thrombosis (28, 29). For MASLD patients, this heightened risk of thrombosis directly impacts the incidence of cardiovascular events and significantly increases the likelihood of cardiovascular disease-related mortality.

The subgroup analysis further strengthens the robustness of our results. However, some of the findings from the subgroup analysis are particularly interesting. The subgroup analysis revealed that male patients with MASLD face a higher death risk, which is consistent with recent evidence indicating that male patients with MASLD have high cardiovascular death risks (30, 31). This finding underscores the importance of considering gender differences in cardiovascular risk assessment, as males may be more susceptible to the negative cardiovascular outcomes associated with MASLD. In addition, our analysis showed that MASLD patients with pre-existing hypertension, diabetes and CVD had a relatively low risk of death due to elevated UACR. This may be due to more aggressive management of these disorders in clinical practice. People with high blood pressure and diabetes are often closely monitored and treated with medications that reduce cardiovascular risk, which may mitigate adverse effects on cardiovascular health. Given its simplicity and ease of measurement in clinical settings, UACR can be incorporated into routine cardiovascular risk stratification frameworks as a criterion for identifying high-risk patients. For individuals with elevated UACR, more proactive interventions may be warranted, such as enhancing liver metabolic function and alleviating kidney burden, in order to reduce the incidence of cardiovascular diseases.

To our knowledge, this is the first analysis to evaluate the utility of UACR in assessing mortality risk among patients with MASLD. By focusing on this theme, our research provides valuable insights into the prognostic value of UACR as a biomarker for MASLD -related outcomes. However, the study also has several important limitations that warrant discussion. Due to the lack of standard imaging techniques, such as ultrasound or transient elastography, to diagnose hepatic steatosis and assess liver disease severity, we relied on the USFLI as a surrogate diagnostic tool. While USFLI is a validated marker, it has limitations in sensitivity and specificity compared to direct imaging methods, potentially introducing misclassification bias in the diagnosis of MASLD and its severity. Due to the stringent exclusion criteria applied in our study, a considerable number of participants were excluded. These criteria were necessary to ensure data quality and address confounding factors but may have introduced selection bias. Despite adjusting for multiple covariates, residual confounding by unmeasured factors (e.g., genetic predispositions, dietary habits, or physical activity) cannot be ruled out.

## 5 Conclusion

In summary, higher levels of UACR within the normal range are closely associated with poor prognosis in patients with MASLD. These findings underscore the importance of monitoring and managing UACR levels in MASLD patients, as well as the necessity of considering UACR in cardiovascular disease risk assessment.

## Data Availability

The original contributions presented in the study are included in the article/Supplementary material, further inquiries can be directed to the corresponding authors.
