# Supplementary material for: Association between urinary albumin-to-creatinine ratio and all-cause and cardiovascular-cause mortality among MASLD: NHANES 2001–2018
Source: Front Nutr. 2025 May 9;12:1528732. doi: 10.3389/fnut.2025.1528732 (PMC12098063; doi:10.3389/fnut.2025.1528732)
Supplement: Supplementary file 1 [file Table_1.docx]

**Association between** **urinary albumin-to-creatinine ratio and all-cause and cardiovascular-cause mortality among MASLD: NHANES 2001-2018**

**Zhengjin Wang^1^, Zhangxin Chen^1,3^, Ruijie Zhang^1^, Hanxu Zhuang^2^**

**1 Zhangzhou Health Vocational College, Zhangzhou, 363000, Fujian Province, China**

**2 Department of Gastroenterology, Zhangzhou Affiliated Hospital of Fujian Medical University, Zhangzhou 363000, Fujian Province, China**

**3 Department of Spine Surgery, Renmin Hospital of Wuhan University, Wuhan, 430060, Hubei Province, China**

**The detailed of definitions:**

**Diabetes was defined as(1)** doctor told you have diabetes, glycohemoglobin HbA1c (%) >= 6.5, fasting glucose (mmol/l) >= 7.0, random blood glucose (mmol/l) >= 11.1, two-hour OGTT blood glucose (mmol/l) >= 11.1, Use of diabetes medication or insulin.

**Hypertension was defined as(2):** systolic blood pressure (BP) ≥ 130 mmHg, diastolic BP ≥ 80, or currently taking BP medications.

**Smoking status was defined as(3)**: never smokers (less than 100 cigarettes in their lifetime), former smokers (at least 100 cigarettes in their lifetime but quit in the past 12 months), and current smokers (at least 100 cigarettes in their lifetime and currently smoking).

**eGFR** was calculated by the creatinine-based CKD Epidemiology Collaboration 2009(4).

1. Yuan S, Song C, Zhang R, He J, Dou K. Dietary Inflammation Index and Its Association with Long-Term All-Cause and Cardiovascular Mortality in the General US Population by Baseline Glycemic Status. Nutrients. 2022;14(13).

2. Liu J, Yi SS, Russo RG, Horowitz CR, Zhang D, Rajbhandari-Thapa J, et al. Trends and disparities in prevalence of cardiometabolic diseases by food security status in the United States. Nutr J. 2024;23(1):4.

3. Zhang J, Cao Y, Mo H, Feng R. The association between different types of physical activity and smoking behavior. BMC Psychiatry. 2023;23(1):927.

4. Levey AS, Stevens LA. Estimating GFR using the CKD Epidemiology Collaboration (CKD-EPI) creatinine equation: more accurate GFR estimates, lower CKD prevalence estimates, and better risk predictions. Am J Kidney Dis. 2010;55(4):622-7.
